# Evolution, Insular Restriction, and Extinction of Oceanic Land Crabs, Exemplified by the Loss of an Endemic *Geograpsus* in the Hawaiian Islands

**DOI:** 10.1371/journal.pone.0019916

**Published:** 2011-05-16

**Authors:** Gustav Paulay, John Starmer

**Affiliations:** Florida Museum of Natural History, University of Florida, Gainesville, Florida, United States of America; University of Plymouth, United Kingdom

## Abstract

Most oceanic islands harbor unusual and vulnerable biotas as a result of isolation. As many groups, including dominant competitors and predators, have not naturally reached remote islands, others were less constrained to evolve novel adaptations and invade adaptive zones occupied by other taxa on continents. Land crabs are an excellent example of such ecological release, and some crab lineages made the macro-evolutionary transition from sea to land on islands. Numerous land crabs are restricted to, although widespread among, oceanic islands, where they can be keystone species in coastal forests, occupying guilds filled by vertebrates on continents. In the remote Hawaiian Islands, land crabs are strikingly absent.

Here we show that absence of land crabs in the Hawaiian Islands is the result of extinction, rather than dispersal limitation. Analysis of fossil remains from all major islands show that an endemic *Geograpsus* was abundant before human colonization, grew larger than any congener, and extended further inland and to higher elevation than other land crabs in Oceania.

Land crabs are major predators of nesting sea birds, invertebrates and plants, affect seed dispersal, control litter decomposition, and are important in nutrient cycling; their removal can lead to large-scale shifts in ecological communities. Although the importance of land crabs is obvious on remote and relatively undisturbed islands, it is less apparent on others, likely because they are decimated by humans and introduced biota. The loss of *Geograpsus* and potentially other land crabs likely had profound consequences for Hawaiian ecosystems.

## Introduction

Isolation leads to disharmonic biotas on islands, because poor dispersers are absent or under-represented relative to good dispersers [1], [2]. Low starting diversity and absence of various major taxa generally lead to ecosystems with fewer types of biotic interactions than on continents. This in turn provides ecological release for some colonizers, while preventing the establishment of others. Some strong ecological interactors, such as mammals and ants, are characteristically underrepresented on or absent from many oceanic islands. Terrestrial flightless mammals do not naturally extend east of the Solomon Islands in the Pacific, while ants appear to have been naturally absent from the Hawaiian, and possibly other remote, island groups [3], [4], but see [5]. The lower intensity of some biotic interactions on oceanic islands has striking consequences. First, islands can be “safe places” [6] for taxa that cannot survive in the face of biotic interactions characteristic of continental settings. For example several land snail and insect families, as well as species of wide-ranging sea birds, are restricted to islands [7], [8]. These taxa appear to be especially vulnerable and suffer massive extinctions following introduction of continental predators [8], [9], [10]. Second, ecological release on islands has fostered macroevolutionary transitions – the invasions of novel adaptive zones. Examples include evolution of terrestrial larval habits by damselflies in the Hawaiian, New Caledonian, and Pohnpeian faunas [11], [12], raptorial carnivory in Hawaiian caterpillars [13], and colonization of land by Caribbean truncatellid snails [14] and Jamaican sesarmid crabs [15]. Third, the low initial diversity of insular biotas has fostered diversification, thus evolutionary radiations are a hallmark of island life: Galápagos finches and Hawaiian fruit flies are celebrated examples [16], [17].

Ecological distinctiveness of oceanic island habitats is reflected in distributional patterns: many taxa are distributed widely among, but restricted to, oceanic islands. For example, while species of achatinellid land snail species have restricted ranges, the family extends from the Juan Fernandéz Islands near South America, to Lord Howe Island near Australia, to the Ogasawara Islands near Japan, and to oceanic islands in the Indian Ocean, yet is absent (except for recent human-facilitated introductions) from the four neighboring continents [18]. Similar wide ranges and oceanic restriction characterizes the land snail families Endodontidae and Partulidae, as well as the beetle family Proterhinidae [7]. Many sea bird species are likewise widespread across one or several ocean basins, yet restricted to islands [8]. Oceanic restriction is also common among shallow, benthic marine species with good dispersal powers, such as hermit crabs, lobsters, and gastropods, a pattern long noted [19], [20], [21] and debated [22], [23], [24], [25].

Oceanic restriction is especially striking among “land crabs” [26]. Level of terrestriality varies substantially among crabs, from species that live strictly intertidally to those that have lost all connections with the sea. In this paper we define land crabs as species that live inland of the supratidal fringe, in coastal vegetation, not including mangroves. We exclude ghost crabs (*Ocypode*), as they are primarily beach inhabitants, although some species enter vegetated zones at beach margins. We include both anomuran and brachyuran crabs. Most land crab species in remote Oceania (Polynesia, Micronesia, and island Melanesia) have wide ranges (all have marine larvae), yet are restricted to islands. Of the 18 or so land crab species widespread in remote Oceania, only one is recorded from the Australian mainland, yet 13 occur on adjacent offshore islands (Table 1) [27]. All four major families of oceanic land crabs include species with such distributions. The omnivorous, terrestrial Coenobitidae (Anomura), comprised of terrestrial hermit crabs (*Coenobita*) and the shell-less coconut crab (*Birgus*), “skip” Australia except for the endemic *Coenobita variabilis*. The conspicuous, large-bodied, detritivorous to omnivorous, and often strikingly abundant land crabs of the family Gecarcinidae (Brachyura) follow a similar pattern: only *Cardisoma carnifex* is known from mainland Australia. The Sesarmidae (Brachyura), a diverse group with a broad range of terrestrial adaptations, has repeatedly invaded land; the three terrestrial species known in Oceania appear to be absent in continental Australia, although others occur there. Among the Grapsidae (Brachyura) only some *Geograpsus* qualify as land crabs (as defined here); this predominantly predatory genus is also absent from the Australian mainland. Peter Davie, who compiled the ABIF [27] crustacean checklist, noted (in litt. 12 April 2004) that “as far as I can find there are no records of *Geograpsus* from the mainland Australia. The catalogue was primarily based on literature, but I have no *Geograpsus* in the QM from northern Australia. … My impression of *Geograpsus* is that it has a mostly insular distribution avoiding the continental land masses, rather like the many gecarcinids and *Birgus*. Perhaps it is the result of predator pressure?”

**Table 1:** Distribution of Oceanic land crabs.

Taxon	Australia	Aus Is	IO	Hawaii
**Coenobitidae:**				
*Birgus latro* (Linnaeus, 1767)	no	yes	yes	no
*Coenobita brevimanus* Dana, 1852	no	yes	yes	no
*Coenobita cavipes* Stimpson, 1858	no	no	yes	no
*Coenobita perlatus* Milne Edwards, 1837	no	yes	yes	no
*Coenobita rugosus* Milne Edwards, 1837	no	yes	yes	no
*Coenobita spinosus* Milne Edwards, 1837	no	no	yes	no
**Gecarcinidae:**				
*Cardisoma carnifex* (Herbst, 1796)	yes	yes	yes	no
*Discoplax hirtipes* (Dana, 1851)	no	yes	yes	?
*Discoplax longipes* A. Milne Edwards, 1867	no	no	no	no
*Discoplax rotunda* (Quoy & Gaimard, 1824)	no	yes	yes	?
*Gecarcoidea lalandii* Milne Edwards, 1837	no	yes	yes	no
*Epigrapsus notatus* (Heller, 1865)	no	no	yes	no
*Epigrapsus politus* Heller, 1862	no	yes	yes	no
**Sesarmidae:**				
*Chiromantes obtusifrons* (Dana, 1852)	no	yes	yes	yes
*Labuanium rotundatum* (Hess, 1865)	no	no	yes	?
*Metasesarma obesum* Dana, 1851	no	yes	yes	no
**Grapsidae:**				
*Geograpsus crinipes* (Dana, 1851)	no	yes	yes	yes
*Geograpsus grayi* (Milne Edwards, 1853)	no	yes	yes	no

List of crabs that extend inland sufficiently to be regularly encountered in coastal forests other than mangroves in Pacific Far Oceania. The occurrences of these species in Australia, islands near Australia (Aus Is) (based on ABIF, 2004), Indian Ocean islands (IO), and Hawaii are indicated based on literature records.

Oceanic land crabs are similarly under-represented in Southeast Asia, although the ranges of all but one of the 18 species listed straddle the area. Peter Ng (in litt. 25 March 1998) noted that *Geograpsus* is absent “in SE Asia per se. It occurs only on islands or peninsulas in areas washed by more oceanic waters. I have caught these guys in southern Taiwan and adjacent islands though! They are apparently affected badly by human activities, especially pests. In one island in Taiwan, their population is decreasing, possibly because of human activities. They are very vulnerable when they move *en masse* to sea to spawn. I suspect like most island crabs (e.g. many gecarcinids and *Birgus*), they are vulnerable to predators, native or introduced at some stage of their life. I believe in many cases, it is the young which are very vulnerable to things like rats, civets, squirrels, monitors etc. and the pressure of these predators on the juveniles forces them to small islands where such predators are absent.” Similarly, in their massive treatise on Chinese crabs, Dai & Yang [28] record *Geograpsus grayi* only from Taiwan, and *Geograpsus crinipes* only from Xisha Island (Paracel Islands), in China. Similarly Sakai [29] records *G. crinipes* only from offshore islands around Japan, and *G. grayi* mostly from offshore islands except for records from Kagoshima and Tosa Bay.

Oceanic land crabs are also under-represented on the African mainland, although diverse and common in the Seychelles and other Indian Ocean islands. Only two records of *G. crinipes*, and single records each of *Coenobita perlatus*, *Coenobita brevimanus*, and *Discoplax rotunda* are known from Africa; “their extreme rarity along the East African coastline confirms their insular character” [30]. Among gecarcinids, *Cardisoma carnifex* is the only species common on the African mainland, as in Australia [26], [31].

While many land crabs are restricted to islands, they are dominant members of the terrestrial biota there, with major ecological impacts. They can be important predators on young birds, invertebrates and seeds, affect seed dispersal, control litter decomposition, and are important in nutrient cycling [32], [33], [34], [35] (see below). Loss of land crabs can lead to large-scale shifts in community structure [35], [36].

Almost all tropical islands are occupied by land crabs; in the tropical Pacific only the Hawaiian Islands “lack” them. However this is a secondary phenomenon, as land crabs were present in Hawai'i before human colonization. Here we describe an abundant, highly terrestrial, large, presumably carnivorous-omnivorous, extinct land crab, and review records of other terrestrial and semi-terrestrial decapods recorded from these islands. Fossils of this species have been known for many years, but their identity has not been clearly established [37]. We also consider the evolutionary origin of this land crab, which apparently was endemic to the Hawaiian Islands. Finally we explore the origin and consequences of some macroevolutionary and macroecological biases associated with land crabs and their relatives.

## Results

### Systematics

#### *Geograpsus* Stimpson, 1858

Four species of *Geograpsus* are currently recognized: *G. lividus* in the Atlanto-East Pacific, and *G. stormi*, *G. crinipes* and *G. grayi* in the Indo-West Pacific region [38]. They can be arranged in a series reflected both in morphology and habitat, with the morphologically and ecologically very similar, supratidal *G. lividus* and *G. stormi* at one end, mostly coastal *G. crinipes* in the middle, and most terrestrial *G. grayi* at the other end. Increased terrestriality is accompanied by greater inflation and posterior narrowing of the carapace, as the branchial chamber becomes modified for air breathing. Remarkably, three species are represented by material from Hawaiian Islands.

#### *Geograpsus severnsi* new species

**Figure 1:**
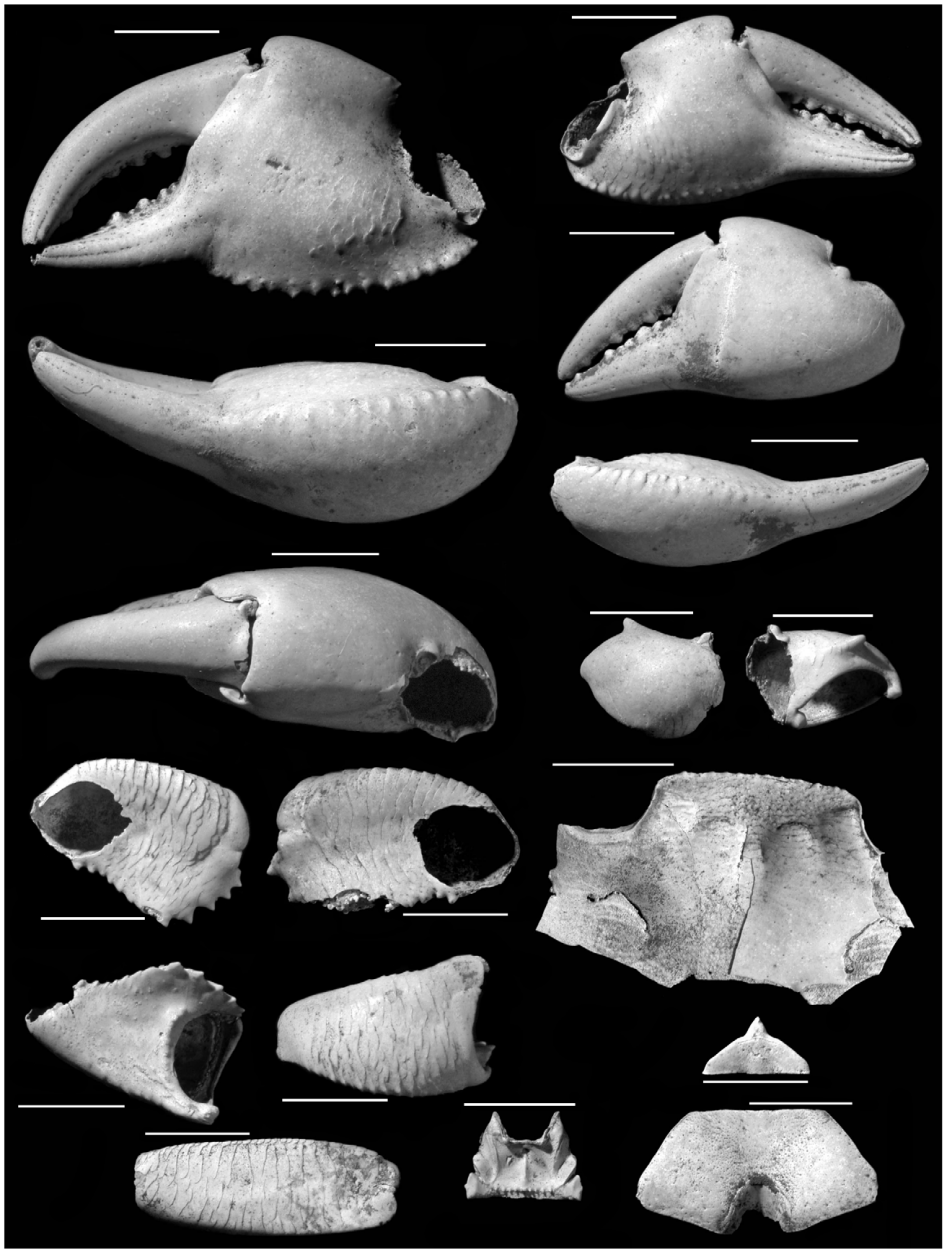
*Geograpsus severnsi*, holotype male (USNM 539,738). Scale: 10 mm.

**Figure 2:**
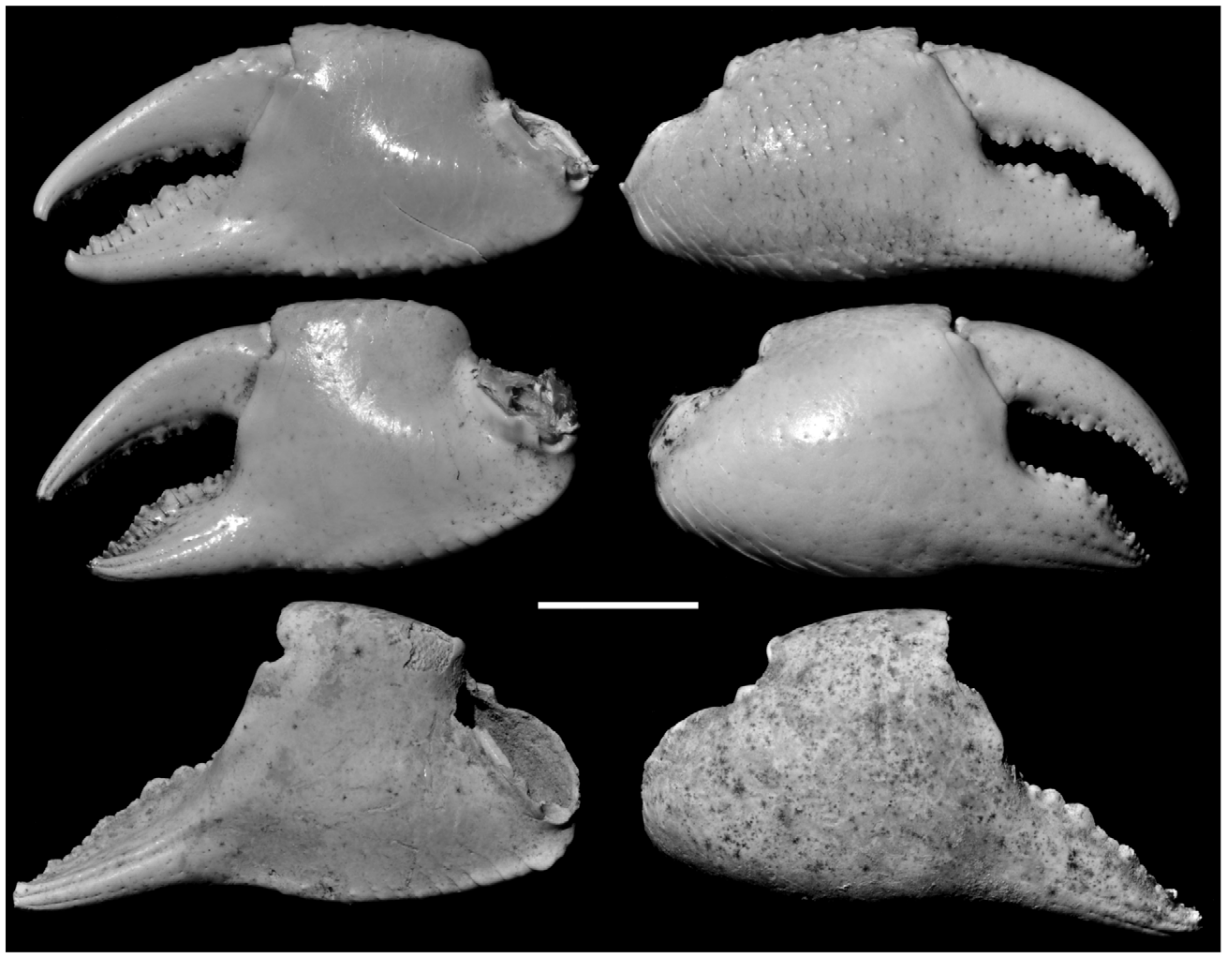
Right chelae of *G. crinipes* (top; UF AR 2166), *G. grayi* (middle UF AR 2381), and *G. severnsi* (bottom, USNM GS-2). Scale: 10 mm.

**Figure 3:**
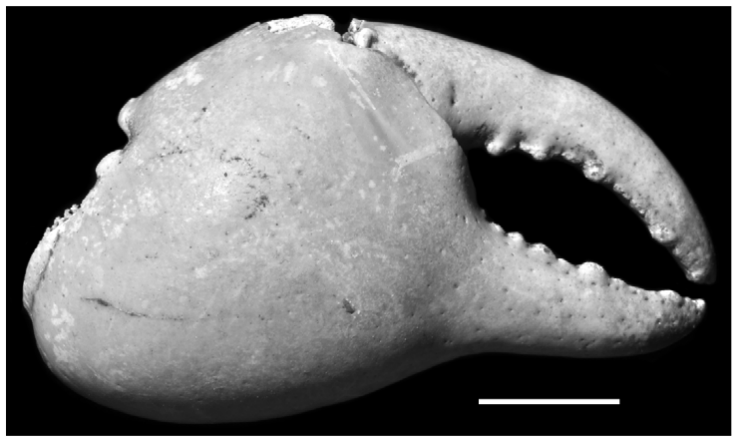
*Geograpsus severnsi*, a large major chela (USNM GS-3). Scale: 10 mm.

**Figure 4:**
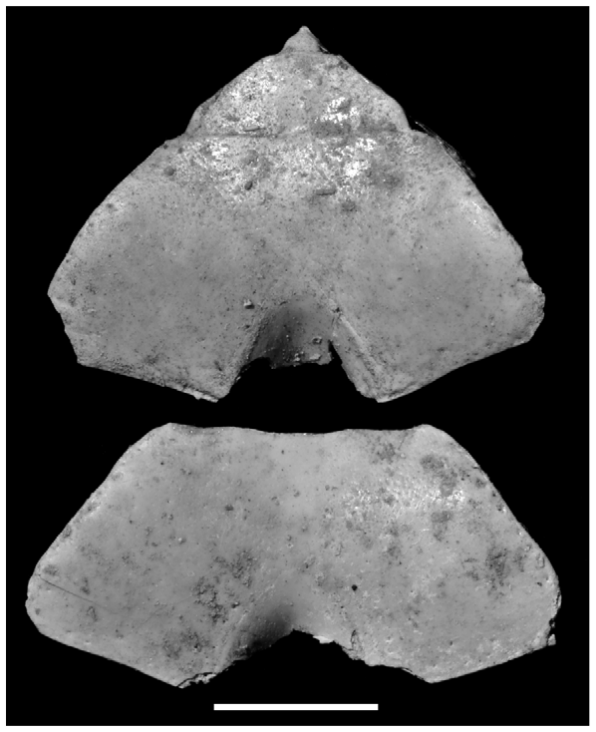
*Geograpsus severnsi*, male (top; UF IP 168,821) and female (bottom; UF IP 168,822) 4^th^ (and 5^th^ – top) sternites. Scale: 10 mm.

**Figure 5:**
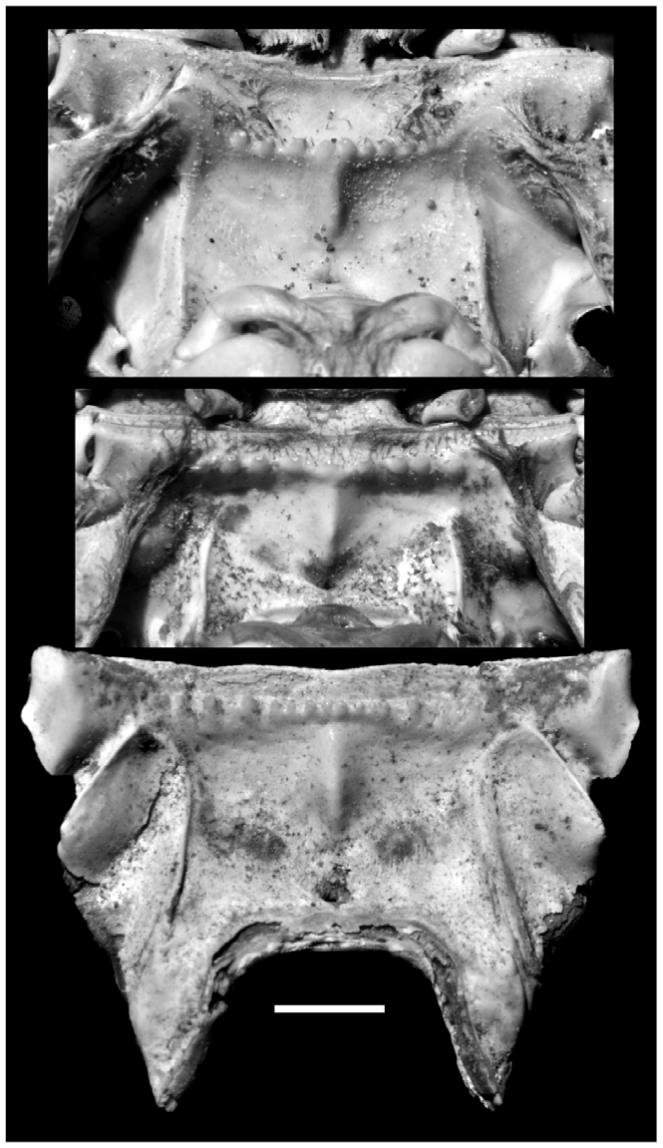
Endostome of *G. crinipes* (top; UF AR 2166), *G. grayi* (middle UF AR 2381), and *G. severnsi* (bottom USNM GS-12). Scale: 2 mm.

Figures 1, 2, 3, 4, 5

- land crab: Howarth, 1990 [37], [39], Giffin, 2003 [38]

- *Geograpsus* species: Burney et al., 2001 [40]

- *Geograpsus* aff. *geayi* [sic]: Burney & Kikuchi, 2006 [41]

#### Material examined

Holotype: USNM 539,738: Maui: Pu'u Naio Cave, 305 m elevation, Quadrat W15.0–15.5, Unit III G. 4.V.1988. H James. 2 sets of cheliped dactyli, propodi, carpi, and meri, pieces of ambulatory legs including meri, portion of carapace, endostome, 4^th^ and 3^rd^ thoracic sternites, pterygostomial plates; presumably from a single male specimen (Fig. 1)

#### Paratypes

All from Maui, Pu'u Naio Cave. USNM 539,739: W17.0–17.5, Unit III I. 22.VI.1988. H James. 1 chela, 1 fixed finger. USNM 539,740: W15.0–15.5, Unit IV F I. 9.V.1988 H James. 1 dactyl, 1 sternite, 1 endostome, chelar merus, carapace fragments. USNM 539,741: W16.5–17.0, Unit III I, 16.III.1988. H James. Male 4^th^ sternite. UF IP 168,821: 1984. M Severns. male 4^th^ and 5^th^ sternites. UF IP 168,822: 1984. M Severns. female 4^th^ and 5^th^ sternites. UF IP 168,823: 1984. M Severns. male 4^th^ and 5^th^ sternites.

#### Non-type

Kaua'i: USNM 539,742: Pa'a sand dunes at Poipu. 25.V.1988, S Olson, Gage, P Fiene, M Severns. 2 partial carapaces, 3 articulated chelipeds. USNM 539,743: Kealia, 26.V.1988. P Fiene, M Severns. 2 claws, 1 dactyl, 1 fixed finger. UF IP 168,827: Makawehi Dunes, S Kaua'i coast, 21°52′51″N, 159°26′03″W, late Pleistocene to early Holocene, lithified carbonate dunes. 20.IX.1997. D Burney, S Olson. 1 claw.

O'ahu: USNM 539,744: Barber's Point: Bishop Museum site 50-0a-B6-22, sqM4, TP1 extension, layer II. 4.IV.1980. 1 claw, 4 chelar dactyli.

Moloka'i: USNM 539,745: Mo'omomi Dunes, west end of old sand mine. 4.VI.1998. 1 chela. S Olson, J Aidem, P Hearty. USNM 539,746: Mo'omomi Dunes, 5-10-11. 29.VII.1990. H James, Aidem, T Stafford, Hume. 2 chelae, 1 chelar propodus.

Maui: USNM 539,747: Waiehu, sand dunes being sand-mined at “Leisure Estates”. 1.VI.1988. S Olson. various claw pieces. USNM 539,748: Lua Lepo, D1W, W1/2 Unit 5. 2 claw fragments, carapace fragments. UF IP 168,824: Pu'u Naio. 1984. M Severns. Pterygostomian fragments. UF IP 168,825: Pu'u Naio. 1984. M Severns. 1 claw dactyl. UF IP 168,826: Maui: Pu'u Naio. 1984. M Severns. Carapace pieces.

Hawai'i: USNM 539,749: Point caves, cave on road towards/into subdivision (near Mark Twain Tree) ca. 5 miles near main highway (Makai), ca. 4.4 mi. NE of S Point Road on left side of dirt road. Crabs from deep in cave, many present. E Paxinos, J Wilcox, R Basona, J Lilly. leg carpus, endostome with associated mandible, carapace fragment, 2 entire claws, 4 cheliped carpi, 2 cheliped meri, 8 ambulatory leg meri, 4 ambulatory leg carpi, 2 male 4^th^ sternites, fragments. USNM 539,750: Makalawena cave. 2.VI.1991. H James, J Earle. 1 male 4^th^ sternite, 2 claws, 2 cheliped carpi, leg fragments; same crab? USNM 539,751: Hawai'i, new cave system, ca. 12m south of and parallel to Owl Cave system, below 213m asl. 3.III.1993. H James, Lopez, Cooper, Griffen, Cobb, B Schaefer. 1 claw, 2 chelar carpi, leg and chelar meri. USNM 539,752: Makalawena cave, 646 m, upslope of first entrance, near third entrance. 2.VI.1991. H James, J Earl. 4 claws, 2 partial chelar meri, 1 dactylus, 1 partial chelar carpus. USNM: 539,753: Kau Kaaluau-Waiohinu Road Cave, 4.5 mi. from HWY 11 through Mark Twain subdivision down slope from Y in road. 1.VIII.1998. E Paxinos, S Olson, H James, B Slikas, B Schaefer. 2 claws, most of male 4^th^ sternite, leg fragments; same crab?

Numerous additional specimens from several of these localities (USNM, UF).

#### Diagnosis

Large *Geograpsus* species with chelae smoother and higher than in congeners, with fixed finger markedly bent downward and with straight cutting surface. Cheliped carpus nearly smooth, with small inner spine; merus with inner margin expanded forming a marked angle, with 4–6 large teeth anterior to angle. Ambulatory meri longer, narrower, and less compressed than in other *Geograpsus* species. Fourth sternites lacking anterior constriction; pubescent as in *G. grayi*.

#### Description

Cheliped (Figs. 1, 2, 3): Chelae large (L = 34.1±8.7 mm; range: 20.4–49.1 mm; N = 37), higher than in other *Geograpsus* species (H/T = 1.58±0.11; range: 1.39–2.00; N = 41), inflated, dimorphic. Large chelae with dactylus conspicuously arched relative to fixed finger; fixed finger extending ventrally at an angle from palm; angle and arising gap between fixed and movable fingers both increasing with size (Fig. 3). Smaller chelae with dactylus slightly arched to straight, with little gap. Cutting margin of fixed finger straight to slightly concave. Palm smoother than in other *Geograpsus* species, becoming smoother with increasing size; dorsal margin bearing 0–3 granules near base; ventral margin marked by row of transversely-elongated granules; outer surface smooth; inner surface with a patch of low, scaly ridges on basal half. Fixed finger with 7 or 8 rows of punctae on inner surface, some rows incised as grooves, and with slightly- to markedly-raised, smooth to granular ridge; sculpture more pronounced in small chelae. Dactylus smooth, with rows of punctae most pronounced in smaller specimens.

Carpus relatively smooth, with a few, low granules and low ridges when small, to completely smooth when large; inner spine smaller than in *G. crinipes* or *G. grayi*.

Merus with well developed, markedly angular flange with toothed margin, bearing 4–6 large teeth distal to, and several, much small teeth basal to, angle. Merus smooth ventrally except near outer border; with a conspicuous transverse ridge distally; ventral portion of ridge low and smooth, dorsal portion higher and granular. Dorsal and outer face of merus with well-developed, imbricating ridges.

Ambulatory legs (Fig. 1): Meri narrower, more elongated, and less compressed than in other *Geograpsus* species; equally broad at proximal and distal ends; distoposterior margin not expanded as in other *Geograpsus* species and lacking spinules; with compressed, well delineated ridge forming dorsal margin; with fine, imbricating ridges along upper and lower surfaces. Carpi typical of genus.

Carapace: (partial specimens available only) Frontal margin of adult straight or slightly convex from anterior aspect; with single anterolateral tooth behind exorbital angle; outer margin of exorbital angle strongly inturned; frontal area with fine, scale-like pattern typical of genus; hepatic and branchial areas with numerous transverse ridges; intestinal area smooth. Posterolateral parts of carapace not preserved among available specimens.

Sternites (Figs. 1, 4): Fourth sternite trapezoidal in shape, lacking anterior constriction of *Grapsus* species and *G. stormi*; with convex anterolateral, and straight anterior margins; densely punctate, thus densely pubescent as punctures hold setae in life; punctae densest in paired tracks that diverge anteriorad around middle of sternite and also abundant along anterolateral margin; punctae generally aligned in short, oblique, postero-lateral to antero-medial, rows. Third sternite typical of genus (Fig. 1).

Endostome (Fig. 5): Upper buccal ridge tuberculate, straight, close to anterior margin of endostome.

#### Etymology

Named for Mike Severns, who discovered the Pu'u Naio Cave on Maui, where most of the material came from; for his long-standing interest in and collections of this species.

#### Distribution

Hawaiian Islands: known from Hawai'i, Maui, O'ahu, and Kaua'i; nearshore to 905 m elevation; Holocene (Table 2).

**Table 2:** Distribution of fossil *Geograpsus* specimens.

Island	Location	Specimens	Species	Elevation
Hawai'i	Honokoa Gulch	Bill Haus, in litt.	*severnsi*	360 m
Hawai'i	Ka'aluau-Waiohinu Road Cave	USNM	*severnsi*	130 m
Hawai'i	Makalawena Cave	USNM	*severnsi*	165–280 m
Hawai'i	Cave system ca. 40 ft south of and parallel to Owl Cave system	USNM	*severnsi*	∼220 m
Hawai'i	South Point cave	USNM	*severnsi*	
Hawai'i	Pu'u Wa'awa'a	Giffin, 2003	*severnsi*	36–957 m
Maui	Kahawaihapapa Cave	USNM	*crinipes*	15 m
Maui	Kiakeana Cave	USNM	*severnsi & crinipes*	73 m
Maui	Lua Lepo Cave	USNM	*severnsi*	808 m
Maui	Pu'u Naio Cave	USNM, UF	*severnsi*	305 m
Maui	Waiehu sand dunes	USNM	*severnsi*	<30 m
Maui	Pu'u Makua Cave	USNM	*severnsi*	1463 m
Moloka'i	Mo'omomi Dunes	USNM	*severnsi*	<20 m
Oahu	ca. 3.6 km N of Barber's point, BPBM site 50-0A-B6-22	USNM	*severnsi*	∼15 m
Kaua'i	Kealia	USNM	*severnsi*	<30 m
Kaua'i	Makawehi	USNM	*severnsi*	<30 m
Kaua'i	Pa'a sand dunes at Po'ipu	USNM, UF	*severnsi*	<30 m

#### Remarks

**Figure 6:**
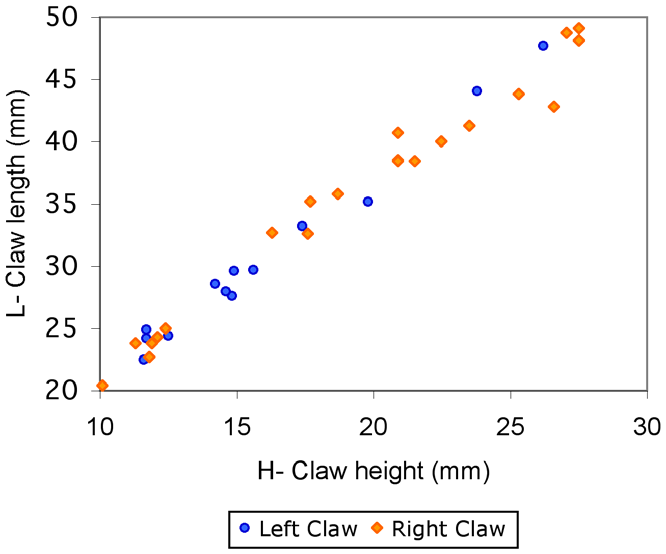
Claw length (L) vs. height (H) in right (orange diamonds) and left (blue circles) claws of *G. severnsi*.

In all the three specimens examined with associated claws the right claw is the major one. Among all claws examined there is a strong bias toward larger size in right claws (Fig. 6).

The fourth sternites of males have a more strongly demarcated and narrower depression than those of mature females for accommodating the telson. This is also expressed in the meristic BW/S ratio, which is >2 in all male and <2 in all female *Geograpsus* species (Table 3). Fossil sternites of *G. severnsi* clearly show this sexual dimorphism and provide a means of sexing the population. Immature females have sternites similar to that of males. In *G. grayi*, females develop mature sternite and abdomen morphology at ca. 6 mm anterior (TW) and 10 mm posterior (BW) sternite width (e.g., UF 4987). The smallest fossil *G. severnsi* sternites are 9.5 mm/20 mm (TW/BW) wide, with two clearly female sternites 10.8 and 10.9 mm (TW) wide. Thus it is likely that all fossil sternites (TW = 12.4+/−1.9, range: 9.5–17, N = 28; TW = 23.9+/−3.0, range: 20–29.5 mm, N = 18) are from mature individuals and thus can be used to sex the crabs. Furthermore, all sternites complete enough to allow measurement of BW and S (N = 17) had male shapes and BW/S ratios appropriate for male *Geograpsus* species(Table 3). According to shape and morphometrics, of 31 sternites examined, 28 were from males and 3 from females.

**Table 3:** Meristics of sternum.

Species	Sex	BW/S	SD	Min	Max	N
*crinipes*	male	2.32	0.12	2.02	2.47	12
*crinipes*	female	1.75	0.10	1.64	1.92	7
*grayi*	male	2.29	0.66	2.20	2.40	14
*grayi*	female	1.81	0.13	1.66	1.89	3
*stormi*	male	2.23	0.07	2.17	2.33	5
*stormi*	female	1.86	0.01	1.85	1.87	2
*lividus*	male	2.28	0.16	2.17	2.76	12
*lividus*	female	1.79	0.07	1.70	1.94	10
*severnsi*	male	2.62	0.12	2.31	2.82	17
*severnsi*	female	-	-	-	-	-

BW/S: mean of sternum width (BW) and posterolateral margin width (S) ratio; SD: standard deviation; Min: minimum value, Max: maximum value, N: number measured.

*Geograpsus severnsi* appears to be an offshoot of *G. grayi*, a hypothesis supported by overall morphological resemblance, as well as phylogenetic analysis (see below). Notably both species have 1) relatively high and lightly ornamented chelae, unlike the elongated and markedly tuberculate chelae of other *Geograpsus* species, 2) a pubescent fourth abdominal sternite (shown by punctures in fossils), 3) appear to have (preservation of *G. severnsi* carapaces too limited to be certain) a more inflated carapace, with the lateral ridge separating the dorsal and lateral parts of the carapace terminating about halfway as in *G. grayi*, rather than continuing to posterolateral corner as in other congeners, and 4) upper buccal ridge on endostome straight and close to the anterior margin, rather than convex and considerably more posteriorad as in *G. crinipes* (Fig. 5). *Geograpsus severnsi* can be readily differentiated from *G. grayi* by its smoother and higher (Fig. 2) chelae, smoother chelar carpi that bear a smaller inner spine than in *G. grayi*, and relatively straight, rather than arched, cutting surface on the fixed finger. The ambulatory meri of *G. severnsi* are more elongated, less distally expanded, and thicker than in other *Geograpsus* species.

**Figure 7:**
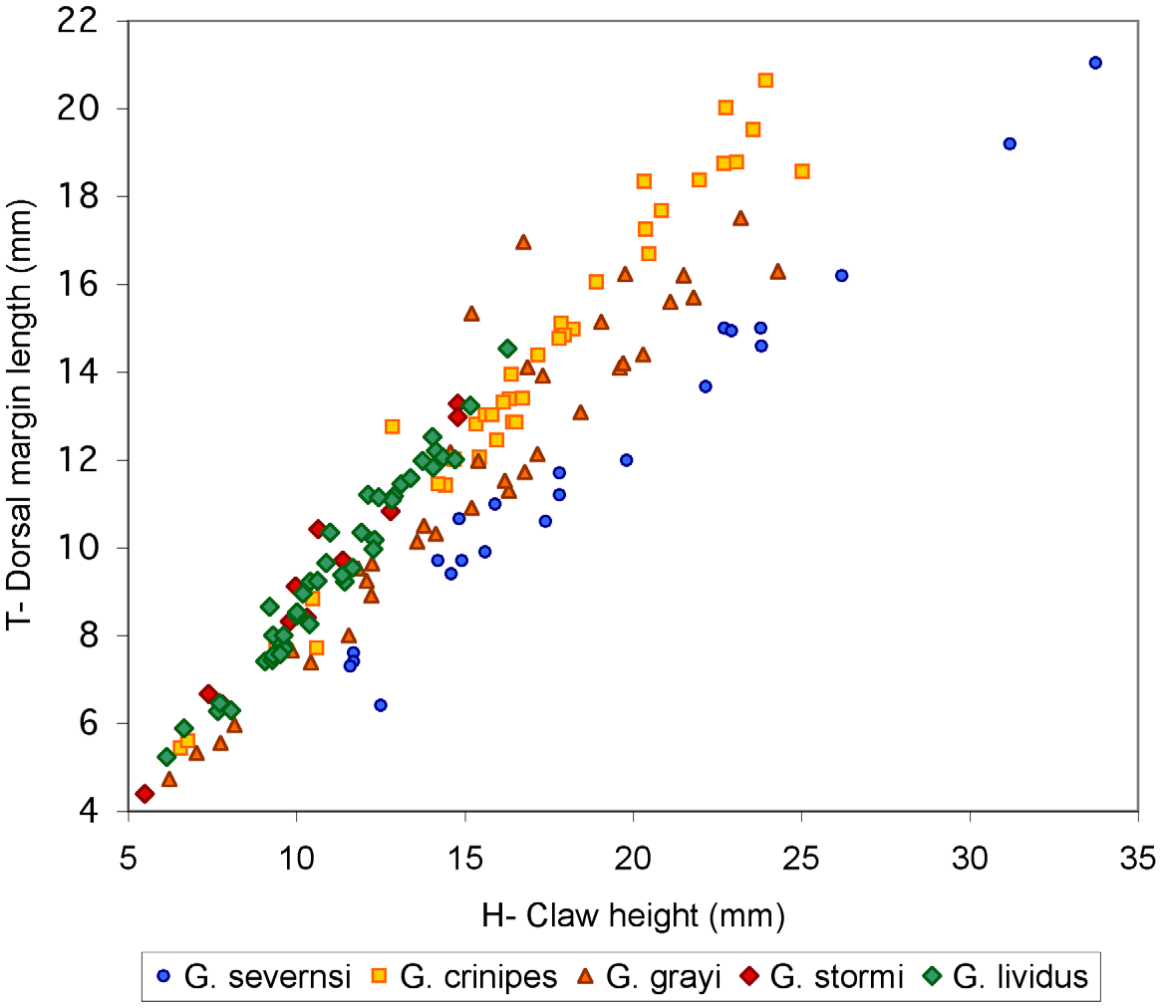
Claw height (H) vs. length of dorsal claw margin (T) in mm.

**Figure 8:**
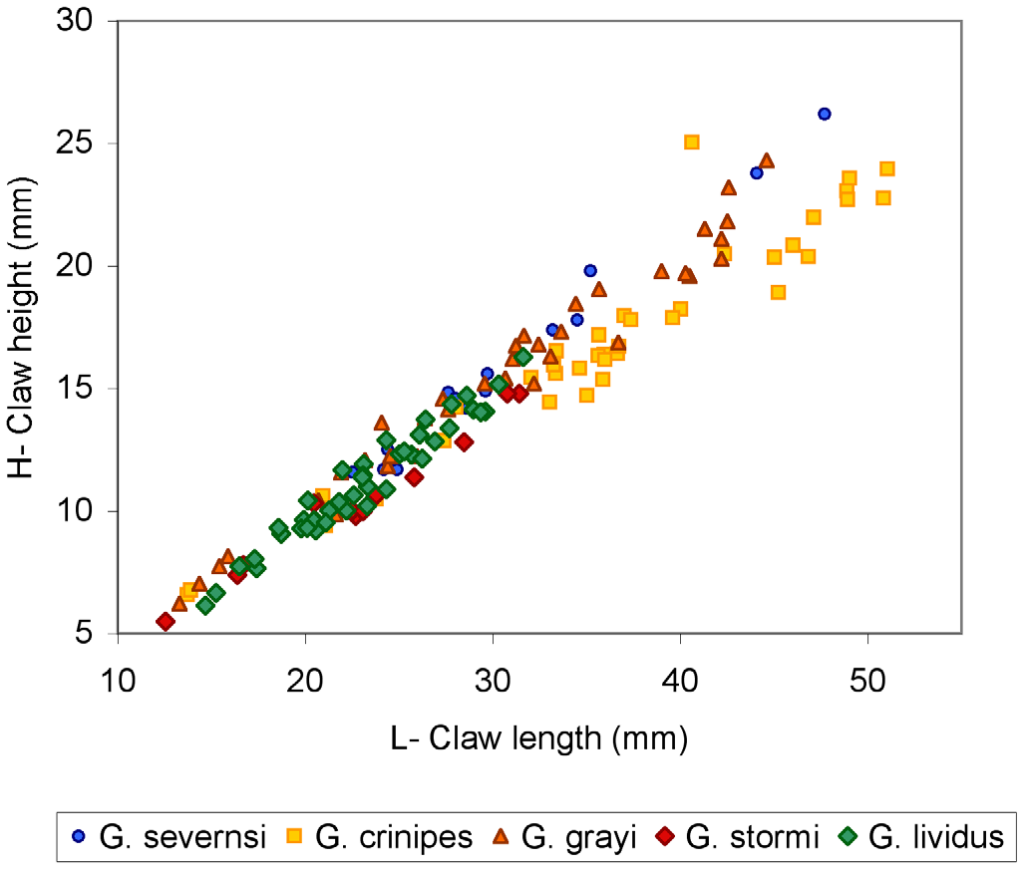
Claw height (H) vs. claw length (L) in mm.

**Figure 9:**
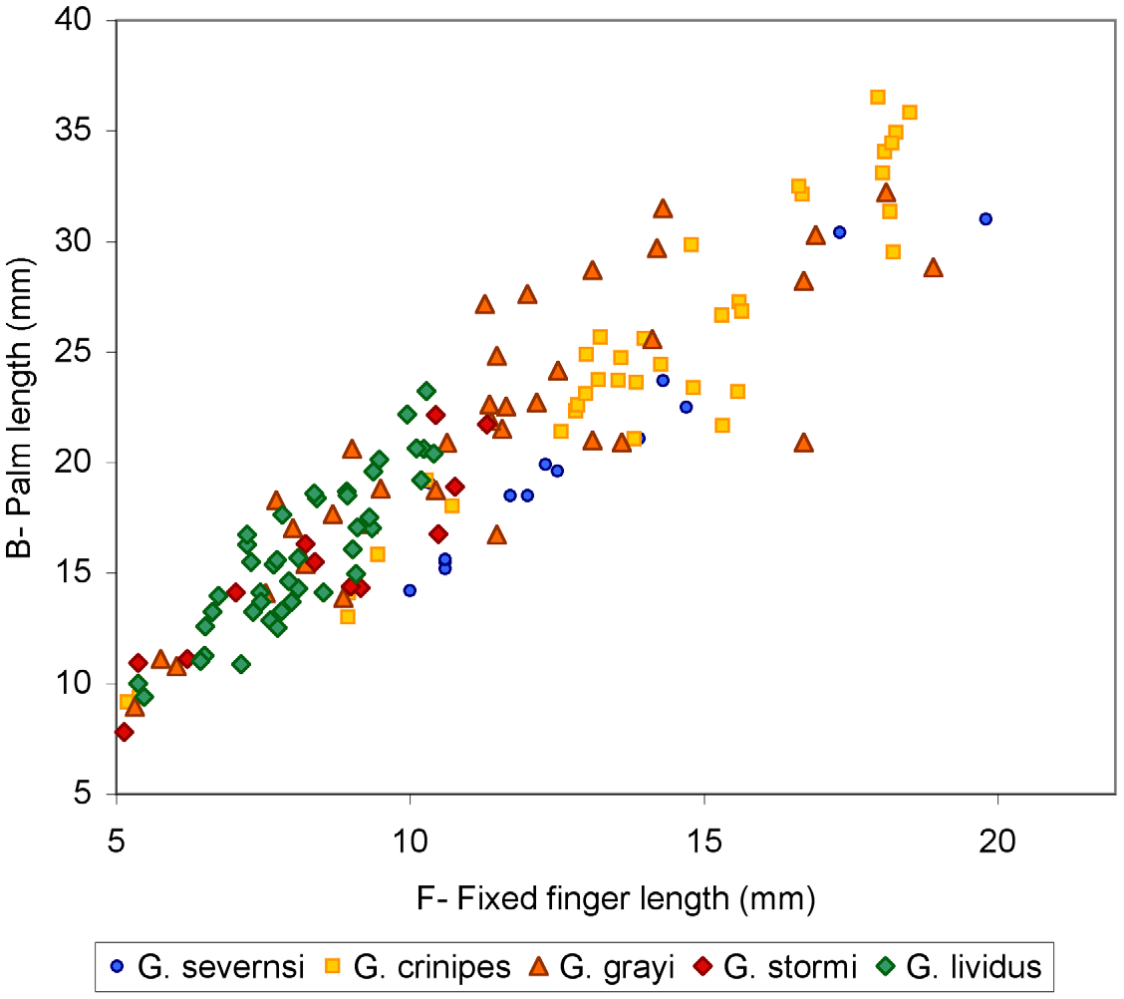
Palm length (B) vs. fixed finger length (F) in mm.

**Figure 10:**
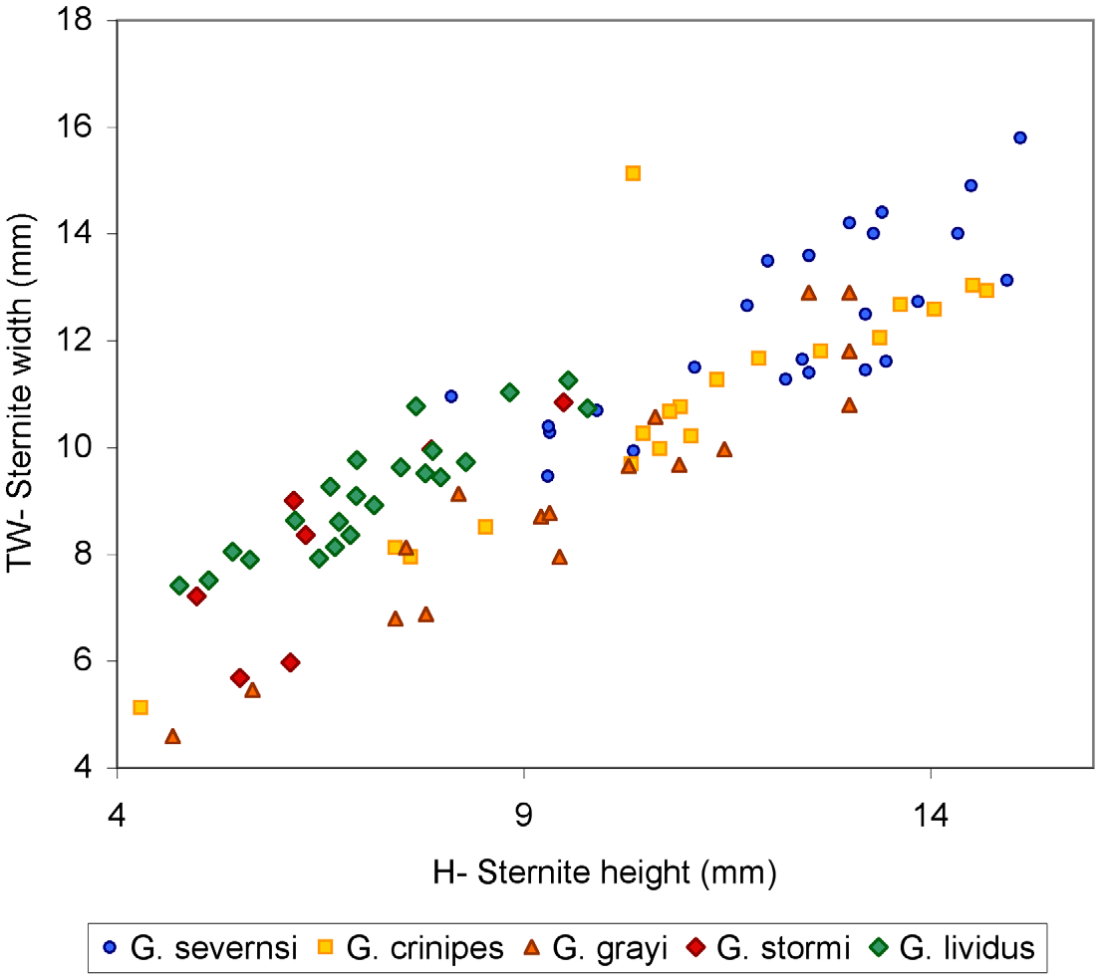
Anterior width (TW) vs. height (H) of 4^th^ sternite in mm.

Claws are the most common fossils and show clear meristic differences among *Geograpsus* species. The claw of *G. severnsi* is proportionately taller (i.e., greater H/L but especially H/T) than of other *Geograpsus* species and bears a relatively longer fixed finger (i.e., lower B/F) than the claw of congeners (Table 4, Figs. 7, 8, 9). The lesser accentuation of the H/L than H/T ratio is the result of the opposite effects of palm heightening and finger lengthening. The 4^th^ sternite of *G. severnsi* also tends to be broader (i.e., greater TW/H) than those of *G. grayi* and *G. crinipes*, but substantially narrower than those of *G. stormi* and *G. lividus* (Table 4, Fig. 10).

**Table 4:** Meristics of *Geograpsus* species.

Species	H/L	H/T	B/F	TW/H
*severnsi*	0.53±0.03; N = 37	1.59±0.11; N = 50	1.53±0.21; N = 37	1.01±0.11; N = 23
*grayi*	0.51±0.03; N = 36	1.33±0.11; N = 36	1.90±0.26; N = 36	0.95±0.08; N = 18
*crinipes*	0.47±0.03; N = 38	1.21±0.06; N = 38	1.76±0.15; N = 38	0.95±0.07; N = 19
*lividus*	0.48±0.03; N = 42	1.20±0.12; N = 42	1.93±0.20; N = 42	1.30±0.12; N = 22
*stormi*	0.45±0.01; N = 12	1.18±0.04; N = 12	1.69±0.18; N = 12	1.27±0.18; N = 7

Mean ± sd; see Fig. 1 for explanation of variables.

**Figure 11:**
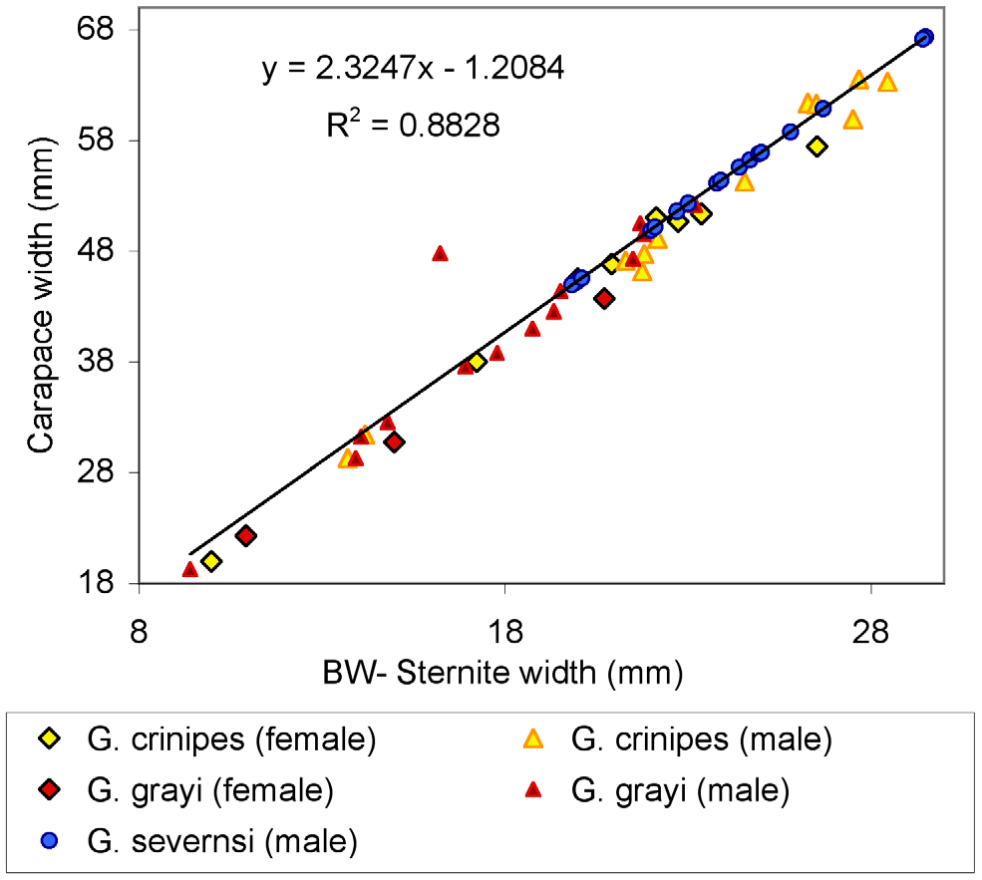
Regression between widths of carapace and posterior width of 4^th^ sternite (BW) in *Geograpsus*, with estimated carapace widths of *G. severnsi* plotted on regression obtained from living species.

*Geograpsus severnsi* may have been the largest species in the genus. There is a tight correlation between width of 4^th^ sternite and width of carapace across all *Geograpsus* species (y = 2.3247x - 1.2084, r^2^ = 0.88; Fig. 11). If we assume this relationship also held for *G. severnsi*, then we can estimate carapace width from size of sternites. Sternites of these fossils slightly exceed the width of even the largest *G. crinipes* (Fig. 11), even though only 18 *G. severnsi* sternites were available, while the sample size for whole *G. crinipes* was much greater and included the largest specimens we have seen. The largest *G. crinipes* measured (UF 2383: Pitcairn I, CW = 63 mm, CL = 55 mm) were close to the maximum recorded size for the species (CL = 61 mm [38]). The estimated carapace width of the largest *G. severnsi* is 66 mm (Fig. 11). Griffin [39] records fossil carapaces up to 55 mm wide. The size of *G. severnsi* is also reflected by its large claws, although claw shapes are too variable to allow an accurate estimation of body size in *Geograpsus* species.

Hawaiian workers have been aware of this species for some time. Frank Howarth sent specimens he collected at 430 m elevation, 5 km inland on Oahu to Fenner Chace, which were identified by Chace (*in litt.*, 20.VI.1975) as *Geograpsus* cf. *crinitus*. A similar query was made by Bill Haus (*in litt.*, 17.III.1980)(Assistant in Invertebrate Zoology at Bishop Museum) to John S. Garth, in which he noted that the fossils “were discovered by John Earle in a burial mound located inside a cave c.a. 1200 ft. above sea level (Honokoa Gulch, Hawai'i).” and that “it is possible that we are dealing with an extinct species”. Garth's reply (24.III.1980), does not add much to Haus' thoughtful analysis.

#### *Geograpsus crinipes* (Dana, 1851)

*Grapsus crinipes* Dana, 1851[42]: 249; 1852 [43]: p. 341, Pl. 21, Fig. 6, Type locality: Hawaiian Islands.

*Geograpsus crinipes*: Kingsley, 1880 [44], Rathbun, 1906 [45], Edmondson, 1946 [46], 1959 [47], Titcomb et al., 1978 [48], Godwin & Bollick, 2006 [49].

#### Material examined

Fossil: USNM 539,761: Maui, Kiakeana Cave, 1.VII.1990. H James, P Fiene, M Severns, F Grady: 2 claws, 10 fixed fingers, 10 dactyli, and fragments. USNM 539,762: Maui, Kahawaihapapa Cave, 50 ft. 22.III.1988. M Severns, P Fiene, S Olson, H James, T Stafford. 2 chelar fixed fingers, 3 chelar dactyli.

Recent: BPBM 5288: Laysan, tide pools. 26.IV.1928. S Ball. 1 female, BPBM-S 255: O'ahu. V.1915. J Stokes. 2 specimens; BPBM-S 3149: O'ahu; Waikiki. 1930. CH Edmondson. 1 specimen; BPBM-S 3677: O'ahu; Black Point. XI.1933. CH Edmondson. Numerous specimens from non-Hawaiian localities (UF, USNM, BPBM).

#### Remarks

Several eroded chelar propodi and dactyli attributable to *G. crinipes* are among crab remains at Kiakeana Cave on Maui, together with remains attributable to *G. severnsi*. Chelae are recognizable as *G. crinipes* by their well-developed tuberculation. Chelar tubercles are absent in *G. severnsi* and *G. grayi*, and finer and largely restricted to the upper half of the chelae in *G. stormi* and *G. lividus*. *Geograpsus crinipes* has also been recorded historically [43], [45], [46], [47], but appears to be quite rare today. The BPBM card catalog lists specimens from Hawai'i, Maui, O'ahu, and Laysan; however only four lots from O'ahu and one from Laysan could be located in the collections. Two of these are of small juveniles, and the other two are also of small animals. Two additional lots, collected in the 19^th^ century by H. Mann and A. Garrett respectively [45], are reported to be at the MCZ. We asked several naturalists active in marine and shore habitats in Hawai'i whether they have ever encountered *Geograpsus* species; most (L. Eldredge, A. Fields, J. Hooper, C. Pittman, C. Zabin) indicated that they have not. Reassuringly, a single specimen was collected in 2004–5 by Scott Godwin on Moloka'i [49], indicating that the species still survives in the area. Interestingly, the photograph taken of the specimen shows a mottled color pattern, unlike the uniform color of *G. crinipes* elsewhere in the Pacific, a difference warranting a phylogeographic examination of the species. Surveys for the species, especially in less impacted habitats, like offshore satellite islands and the Northwest Hawaiian Islands, would be worthwhile.

*Geograpsus crinipes* is widely distributed in the Indo-West Pacific from the eastern Indian Ocean to Easter Island [26], [38], [50]. It is supratidal and ventures into coastal forests. In uplifted karst islands in the South Pacific (Tonga, Niue, Cook Islands, Henderson Island), we have found it to be a characteristic occupant of coastal karst cliffs, where it runs rapidly from just above to many meters above, tide level. It is also common in coastal forests on karstic terrain, to about 100 m inland. Restriction of this species to a <300 m wide coastal belt has been noted in most areas [51], [52], pers. obs., with two notable exceptions. A single specimen was collected by Thor Heyerdahl on Easter Island in a crevice at 250 m elevation [50]. On volcanic Pitcairn Island, the species extends as far as 500 m inland, to 150 m elevation, in moist coastal to interior forest (JS pers. obs.).

*Geograpsus crinipes* was only encountered in the Hawaiian fossil crab material from Kiakeana Cave, at 73 m elevation (H. James, pers. comm.), and Kahawaihapapa Cave at 15 m elevation. These records are substantially more coastal than most *G. severnsi* collections, but also at a substantial elevation compared to other records of the species. Together with the unusual color pattern of Hawaiian crabs, it suggests that *G. crinipes* may be a species complex, with the nominal species potentially restricted to the Hawaiian Islands.

**Figure 12:**
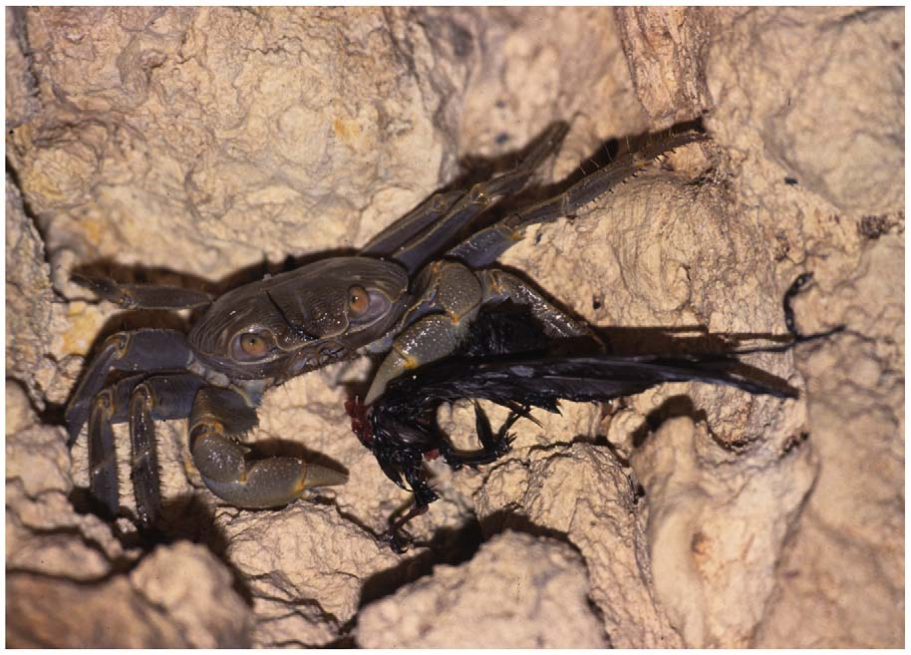
*Geograpsus crinipes* devouring swiftlet, Palau (photo courtesy of Mandy Etpison).

On some remote and relatively undisturbed islands *G. crinipes* can be diurnal, while at other, more disturbed localities (e.g. Guam), it is predominantly nocturnal (GP, JS pers. obs.). In karst it occupies the ubiquitous crevices when hiding. On volcanic islands it does not always burrow, and is found either simply associated with leaf litter (Pitcairn) or on lava outcroppings with crevices (Pitcairn and Tutuila islands). It appears not to burrow on sandy cays, and was associated with leaf litter or fallen trees (e.g., Oeno Atoll and Mañagaha Islet, Saipan). *Geograpsus crinipes* is a voracious omnivore, often preying on other land crabs, including small coenobitids, ocypodids [53], and *G. grayi* [52] (pers. obs.), insects [53], and even birds [54] (Fig. 12).

***Geograpsus lividus*** **(H. Milne Edwards, 1837) complex.**

- *Geograpsus lividus* [45], [46], [47]

- *Geograpsus stormi* [38]

#### Material examined

Recent: MCZ 6107 (Original No 65): Sandwich Islands, Coll. A. Garrett, presented by J.M. Barnard, formalin fixed, 2 males.

#### Remarks

Remarkably, a third *Geograpsus* species is also recorded from the Hawaiian Islands, based on two males collected by Andrew Garrett in the 19^th^ century. They were first listed by Rathbun [45] as *G. lividus*, repeated by Edmondson [46], [47], who noted that “there are no records of the species having been observed in Hawai'i in recent years, and there are no specimens in Bishop Museum.” Banerjee [38] did not examine these specimens, but reassigned them to *G. stormi* based on his recognition of *G. stormi* as the Indo-West Pacific species in the *G. lividus* complex, and assuming an Atlanto-East Pacific restriction to *G. lividus* proper. Although the *G. lividus* complex requires further work because the two species have few morphological differences [38], they are clearly differentiated by color. Thus *G. stormi* has a dark red carapace, while East Pacific and West Atlantic *G. lividus* are tan with dark, variegated patterning.

Banerjee [38] lists 6 characters that differentiate these species; however we were not able to substantiate a number of these as reliable when comparing typical *G. stormi* (UF 10621, etc) with *G. lividus* (UF 8424, 8291, etc). Thus *G. stormi* examined show *lividus*-type ridges at the anterior branchial region of the carapace (Banerjee's character 1); the external maxilliped palps of the two are equally hairy (character 3), and the antero-distal angle of the second walking leg (character 4) in some *G. lividus* we examined are as pointy as illustrated for *G. stormi* by Banerjee. We noted some, but not profound differences in the shape of the upper buccal margin (character 2), mostly as a result of the fusion of the central three teeth on the upper buccal ridge in *G. stormi*, with the resulting larger tooth accentuating the posterior arching of the ridge. The legs of *G. lividus* appear generally shorter, and Banerjee may be correct that this is especially noteworthy for the dactyli (character 5), although we have not quantified this. Colors (character 6) are strikingly different. In addition the carapace of *G. stormi* tends to diverge more posteriorly, while that of *G. lividus* has more subparallel margins. We were not able to confidently assign Garrett's specimens to species. The ridge along the upper border of the buccal cavern are closer to typical *G. lividus* than *G. stormi*, as are the teeth along the anterodistal angle of the second walking legs. The carapace of the larger specimen diverges posteriorly, while that of the smaller is subparallel. As the Hawaiian biota includes some East Pacific elements, the possibility that Garrett's specimens are *G. lividus* remains. However *G. stormi* is known from the adjacent Line Islands (UF 10621). Although distributed across the Indo-West Pacific, *G. stormi* is rather cryptic, typically uncommon, and rarely collected. Thus lack of records since Garrett's [47] (L. Eldredge, A. Fields, S. Godwin, J. Hooper, C. Pittman, C. Zabin, pers. comm.) are less conclusive for this species than for others discussed here, but so is the Hawaiian origin of the material, being a single lot, with little collection information, dating from the 19^th^ century.

This species is restricted to the supratidal fringe, and thus is less terrestrial than other crabs discussed in this paper. Given that *G. severnsi* appears to be an offshoot of the widespread *G. grayi* (see below), all three Indo-West Pacific *Geograpsus* lineages may thus have reached Hawai'i.

### Other Hawaiian land crabs

The only other (marginally) terrestrial crab unambiguously documented from the Hawaiian Islands is *“Chiromantes” obtusifrons* (Dana, 1852) (see [55] for discussion of generic assignment). Described from Maui [43], it ranges to the eastern Indian Ocean [56]. On Guam it lives under rocks and in karstic crevices in sparsely vegetated coastal platforms, up to ca. 50 m inland and ca. 10 m elevation. Edmondson [47] notes it to be intertidal and “even above the high water mark”, suggesting that the Hawaiian population may not be as terrestrial as populations in Micronesia. We have found this species in a supratidal boulder field on O'ahu. Additional observations on its ecology in Hawai'i would be useful.

Five additional species of land crabs (as defined above) have been recorded from the Hawaiian Islands; however all are based on single collections from the 19^th^ century and are questionable. Two species of gecarcinid crabs, *Discoplax hirtipes* (Dana, 1851) and *Discoplax rotundum* (Quoy & Gaimard, 1824) were recorded from O'ahu based on a single lot (MCZ 5769) attributed to H. Mann, 1864. Rathbun [45], Edmondson [57], and Türkay [31] have commented on this lot, comprised of a male and a female specimen. The last author concluded, in his revision of the group, that the male represents *D. hirtipes* and the female *D. rotundum*. We have not reexamined these specimens. We are not aware of any other records of gecarcinids from the Hawaiian Islands, living or fossil, although both species extend well into the central Pacific, reaching eastern Polynesia [58]. If indeed two species are represented in MCZ 5769, then it seems unlikely that this lot originated in the Hawaiian Islands, as the chance that both species would have been recorded but once, by the same person at the same place, seems remote, especially because they have rather different ecology and habitats.

*“Geosesarma” angustifrons* (A. Milne Edwards, 1869) was described from the Hawaiian Islands, but never subsequently collected there [47]. It has also been recorded from the Society Islands [59], [60] and Java [61]. The species belongs to *Sesarmops* Serène & Soh, 1970 (PKL Ng and T Naruse, pers. comm.), a genus of typically stream-associated crabs, a habitat also characteristic of *S. angustifrons* [60] (pers. obs. on Moorea & Raiatea, Society Islands). *Labuanium rotundatum* (Hess, 1865) is another sesarmid that remains poorly documented from the Hawaiian Islands. Edmondson [47] notes “a specimen was collected near [sic] Oahu nearly 100 years ago but, in so far as we know, there are no recent records from this locality.” It is an arboreal crab of coastal forests that is rarely encountered because of its secretive habits. It extends eastward to the Marshall and Tokelau Islands [62], [63] (as *Sesarma (Labuanium) gardineri*). Finally, *“Sesarma” trapezium* Dana, 1852 is known from the holotype supposedly collected in the Hawaiian Islands [46], [47]. Abele [64] considered the limited description to fit the west Atlantic *Armases rubripes* (Rathbun, 1897), a conclusion deemed premature by Bayer [65], but not easily resolved, because the type and only specimen has been lost.

### Phylogenetic analysis

Analysis of fossil species cannot take into account characters widely used in modern brachyuran phylogenetics (G1, G2, male abdomen, vulva, protection of penis, etc.). The following discrete characters were identified that serve to differentiate *Geograpsus* species and are discernible in the fossil material. The character states for each species are summarized in Table 5.

1. Frontal margin of adult: a) straight, b) dorsally convex, c) ventrally convex
2. Fourth thoracic sternite: a) largely glabrous/apunctate, b) mostly glabrous/few punctures, c) densely pubescent/punctate
3. Lateral margin of 4th thoracic sternite: a) straight or convex, b) concave
4. Anterior margin of 4th thoracic sternite: a) sinuous, b) markedly concave, c) straight to slightly concave
5. Outer margin of exorbital angle: a) inturned, b) straight
6. Intestinal region of carapace: a) grooved, b) smooth
7. Merus of 4th pereopod: a) with distal half about same height as basal half, b) with distal half higher than basal half
8. Inner surface of merus of 4th pereopod: a) vertically ridged, b) smooth except near upper margin
9. Merus of 4th pereopod: a) wide, b) compressed
10. Distal ventral margin of merus of 4th pereopod: a) not expanded, b) expanded
11. Claw height: a) low (H/T∼1.2), b) medium (H/T∼1.4), c) high (H/T>1.5), d) very high (H/T>2)
12. Cutting surface of fixed finger in adults: a) straight to slightly concave, b) convex
13. Dorsal margin of claw: a) largely smooth, b) with fine granules, c) with coarse granules, d) with granules and teeth
14. Lateral side of socket for articulation of chelar dactylus: a) grooved, b) jagged, c) smooth
15. Ventral claw surface: a) single axial ridge of granules, b) scattered coarse granules, c) fine oblique ridges, d) coarse oblique ridges
16. Inner face of fixed finger: a) with few or no hairs/punctae, b) with hairs/punctae in row or groove
17. Sculpture on inner surface of palm: a) with strong ridging, b) smooth or with slight ridging, c) with patch of scaly ridges near base
18. Inner, toothed flange of merus of cheliped: a) arcuate, b) angular
19. Vertical ridge on inner face of cheliped merus: a) absent, b) without coarse granules at dorsal end, c) with one coarse granule at dorsal end, d) with several coarse granules at dorsal end
20. Dorsal surface of cheliped carpus: a) with granules or ridges, b) nearly smooth

**Table 5:** Character states.

Sp/character	1	2	3	4	5	6	7	8	9	10	11	12	13	14	15	16	17	18	19	20
*tenuicrustatus*	c	a	b	a	b	b	a	b	b	a	d	a	d	c	d	b	a	a	a	a
*lividus*	a	a	b	c	b	b	b	b	b	b	a	b	b	c	c	a	b	a	b	a
*stormi*	a	a	b	c	b	b	b	b	b	b	a	b	b	c	c	a	b	a	b	a
*crinipes*	b	b	b	b	a	a	b	a	b	b	a	b	c	b	b,d	a	b	a	b	a
*grayi*	a,c	c	a	c	a	b	b	a	b	b	b	b	a,c	c	d	b	b	b	c	a
*severnsi*	a	c	a	c	a	b	a	a	a	a	c	a	a	a,c	a	b	c	b	d	b

**Figure 13:**
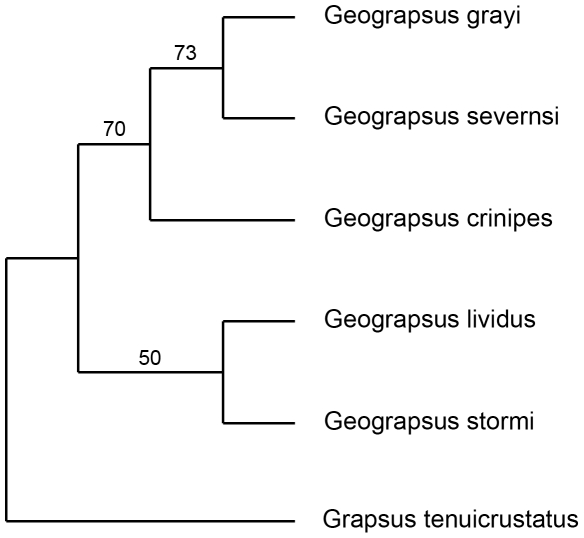
Single most parsimony tree with 10,000 bootstrap replicates for morphological data from all *Geograpsus* species.

An exhaustive search on these characters using the criteria of maximum parsimony algorithm resulted in a single most parsimonious tree (Fig. 13, CI = 0.886, RI = 0.714). Eleven of the 20 characters scored were parsimony informative. The estimated phylogeny shows two clades within *Geograpsus* supported by bootstrap values >70%: *G. lividus*-*G. stormi*, and *G. grayi*-*G. severnsi*.

## Discussion

### Distribution and ecology

*Geograpsus severnsi* was widespread among, but apparently endemic to, the Hawaiian Islands. Fossils are known from Hawai'i, Maui, O'ahu, and Kaua'i. Its sister species, *G. grayi*, ranges from the western Indian Ocean to the neighboring Line and Marshall Islands.

*Geograpsus severnsi* extended further inland than any other *Geograpsus* or other land crab in Oceania (see Table 2 for range of localities and elevations). Fossils are known from nearshore settings to 950 m elevation [39]. Crab remains are abundant at 305 m elevation in the Pu'u Naio lava tube, Maui, located ca. 2 km inland. In the Pu'u Wa'awa'a area of Hawai'i, fossil “land-dwelling crabs were found from 120 to 3,160 feet elevation. Remains were most numerous, however, at about 500 feet elevation” [39]. At 73 m elevation at Kiakeana Cave on Maui, remains of *G. severnsi* are common and co-occur with those of *G. crinipes*, indicating that like *G. grayi*, it overlapped in habitat with *G. crinipes* shoreward. The terrestriality of *G. severnsi* further supports its relation to *G. grayi*, the most terrestrial living grapsid.

It is unlikely that *G. severnsi* had the highly male-biased (8∶1) sex ratio) observed among 4^th^ thoracic sternites (N = 18); rather this suggests preservation bias. It is also unlikely that female 4^th^ thoracic sternites would be less likely to be preserved under the same conditions as male sternites. Rather, the biased sex ratio suggests a difference in where males vs. females were most likely to molt or die. All oceanic land crabs rely on the sea for the development of larval stages, and females undertake coastal migrations to release larvae (e.g. [66]). These migrations as well as the larval release are risky, as they are exposed to a variety of predators first from land and sea, as well as the chance of getting washed away while releasing larvae [66]. We suggest that this is the cause of the biased sex ratio: males, not having to leave the crevices where these deposits formed, tended to die there, whereas females more often died during reproductive migration, at or *en route* to the sea. Similarly biased sex ratio was observed as a result of reproductive migrations in the Ascension Island gecarcinids crab *Johngarthia lagostoma* [67]. This implies that *G. severnsi* had a typical life history for the genus that included a marine larval stage. This provides evidence against the hypothesis that the extinction of *G. severnsi* was driven by a change toward an entirely terrestrial life history, a life history pattern that has evolved in some other terrestrial grapsoids [15], [26].

### Extinction

The occurrence and loss of Hawaiian land crabs has been noted, but the identity of the species previously remained unresolved [37], [40]. All fossils examined were attributable to *Geograpsus*, most to *G. severnsi*, a few to *G. crinipes*. None of the fossils were attributable to other land crabs putatively recorded from the Hawaiian Islands. However the most deposits from which material was examined are located further inland that the habitat range of other potential species. Thus while *Geograpsus* species were clearly well represented in Hawai'i before human arrival, evidence for other land crabs remains equivocal.

There is little doubt that *G. severnsi* is extinct. The species is known only from the Hawaiian Islands, where it extended far inland. The terrestrial biota and ecosystems of the Hawaiian Islands are as well documented as that of any tropical archipelago [68], and no inland crabs have been recorded during historic times. In contrast the coastal *G. crinipes*, much less common in the fossil record than *G. severnsi*, is known from multiple historical specimens, although it appears to be quite rare today. The survival and even occurrence of *G. stormi* is the most poorly constrained.

Abundant *Geograpsus* fossils have been taken from two excavations with well-dated stratigraphic context, which were studied to document changes in the Hawaiian biota and landscape following human colonization: the Pu'u Naio lava tube on Maui [69] and the Māhā'ulepū Caves and Sinkhole on Kaua'i [40]. Deposits at Pu'u Naio extend back to >7,750+/−500 BP across four major stratigraphic units. Unit I is youngest, corresponding roughly to historic times, Unit II dates from the Polynesian expansion, with humans arriving late in Unit III. Most of Units III and IV predate human colonization. The majority of the crab remains we studied are from Unit III, with a few from Unit IV. Cory Pittman (in litt. 11.IV.2004) quantified the abundance of crab fragments at Pu'u Naio and found the last fragments in the basal-most layer of Unit II. Thus at this site *G. severnsi* survived to, but disappeared rapidly after, human arrival. Burney et al. [40], [41] came to a similar conclusion for Māhā'ulepū, where crabs are present in all pre-human layers, show a decrease in size during the early human period, and are absent from more recent strata.

*G. severnsi* appears to be the first documented crab extinction in the Holocene. IUCN's Red List (http://www.redlist.org/search/search-basic.html, accessed 10.IV.2004) records 7 crustacean species as historically extinct, all freshwater taxa. No terrestrial or marine crustaceans have been documented to have become extinct in historic times. That the first documented crab extinction is of a land crab in the Hawaiian Islands is not surprising. The Hawaiian terrestrial biota suffered a veritable mass extinction following human arrival, and this mass extinction continues today. Prehistoric extinctions following the human colonization have been documented in all terrestrial taxa with an appreciable fossil record. Thus over 50% of the Hawaiian bird fauna have gone extinct [69], [70]. Some bird fossils are from the same deposits as the land crab remains reported on here [69], [70], [71]. Predation by humans, introduced rats, pigs, dogs, and habitat destruction, especially of most of the lowland forests, have been implicated in prehistoric extinction of Hawaiian birds, and many additional agents of extinction, such as introduction of avian pox and avian malaria [72], are implicated in historic times [40], [73]. Land snails also suffered catastrophic extinctions, again substantially accelerated during historical times [9], [40], [74].

Oceanic restriction of many land crabs suggests that they are especially vulnerable to continental predators. Although island land crabs remain understudied, they do appear to be more resilient than many other terrestrial taxa. Marine larval stages, wide dispersal capacity, and wide ranges provide rescue mechanisms against population extinction. In contrast, *G. severnsi* was a Hawaiian endemic, although its life history is not known. Marine larval stages have been lost in sesarmid crabs in Jamaica and SE Asia (e.g., [55], [75]). No grapsid crab is known to have evolved such abbreviated development, and the biased sex ratio observed suggests that *G. severnsi* also had marine larvae.

Although oceanic land crabs remain common on many Pacific islands, they appear to be frequently in decline, from a variety of causes. Some are sought after for food, with the very large and long-lived coconut crab most impacted by human predation [76], [77]. Gecarcinids are also often heavily targeted, but at least some species appear to sustain considerable harvesting pressure (pers. obs.). *Geograpsus* species do not appear to be as favored for food as these other terrestrial decapods, and appear to be less commonly harvested [63] (pers. obs.). In the Cook Islands, there is superstition that “killing this crab in the house results in boils because the crab at this time is possessed by the spirit of an ancestor” [78]. Yaldwyn & Wodzicki [63] note that in the Tokelaus *G. crinipes* “are eaten, but are reputed to cause constipation.”

Continental terrestrial predators, most likely mammals, appear to have an especially negative impact on land crabs. The recent introduction of black rats (*Rattus rattus*) to Clipperton Island has resulted in a rapid decline of the abundant *Gecarcinus planatus* there; the same crab suffered substantial decrease in abundance in the first half of the 20^th^ century when feral pigs were introduced to (but subsequently eliminated from) the island [36], [79]. Yaldwyn & Wodzicki [63] noted pigs and humans as the main predators on land crabs in the Tokelaus, where the introduced, but relatively benign, Pacific rat (*Rattus exulans*) was the only rat species present. A devastating loss of gecarcinid crabs is currently unfolding on Christmas Island (Indian Ocean) as a result of the spread of the invasive yellow crazy ant (*Anoplolepis gracilipes*), which readily kills crabs and completely clears areas of forest of these keystone animals [35]. While *Geograpsus* remains common on many less-developed Pacific islands, on more impacted islands, such as Guam it appears to have declined. On Guam *G. crinipes* is now relatively uncommon, while we never saw *G. grayi* during the 1990's when we both lived on Guam, although it is known from neighboring, less-developed islands in the archipelago [80]. Similarly, the nearshore land hermit crab *Coenobita perlatus* has become very uncommon on the main island of Guam, although it remains abundant on offshore Cocos Islet. It is noteworthy that both *G. grayi* and *G. crinipes* were recorded on Mañagaha Island, a small, sandy coral cay lying 2.5 km from Saipan, about a year after the eradication of rats and cats from the island. *G. grayi* has yet to be recorded from Saipan and *G. crinipes* is uncommon.

The agent of extinction for *G. severnsi* remains to be established, although the crab's demise is clearly associated with, and occurred rapidly after, the arrival of humans. Titcomb et al. [48] note that *G. crinipes* (*pai'ea*) was eaten by indigenous Hawaiians, so it is likely that *G. severnsi* was also. Considerable indigenous knowledge accumulates about land crabs on Pacific islands, with detailed understanding of their migration patterns, life cycles, habitat use, etc., and this information is used for harvesting these animals [81].

Polynesian colonists also introduced numerous animals and plants, including pigs, dogs, Pacific rats, jungle fowls, various lizards, insects, and land snails [82]. Rats in particular are known to be important predators on island life [33], although only *R. exulans* arrived with native Hawaiians, and this species coexists with other *Geograpsus* (see above). Major alteration of especially lowland habitats also had devastating affects. Finally, Giffin [39] suggests that the disappearance of large sea bird rookeries, on which the crabs may have partly depended for food, may also be implicated in their extinction. However, the same package of introduced taxa and similar environmental problems were transported to other oceanic islands *en route* to Hawaii and are not known to have led to the extinction of *Geograpsus* or other land crabs there. The highly terrestrial *G. grayi*, the likely sister species of *G. severnsi*, remains common on many islands that have received the same suite of Polynesian introductions. Thus either some unusual combination of factors made pressure on land crabs greater in the Hawaiian Islands than elsewhere, and/or *G. severnsi* was especially vulnerable.

### Macroevolutionary transitions to land

Macroevolutionary transitions between marine and non-marine habitats are uncommon and rarely lead to long-term success in the invaded habitat [83]. Overall, decapod crustaceans have not been particularly successful on land. Nevertheless decapods have become terrestrial on numerous occasions: four families are represented among oceanic land crabs listed in Table 1, several other lineages have evolved onto land elsewhere, and many others show lesser degrees of terrestriality [26]. So if land crabs have evolved so many times, what prevents them from becoming a major player in the terrestrial biome? Predation by terrestrial carnivores, especially vertebrates, is likely important in limiting their success, as are life history ties to the sea, at least during the initial stages of invasion [26], [75]. Planktonic larval stages (zoeae and megalopae) are plesiomorphic for crabs, have rarely been lost in marine crabs, but have been lost in several freshwater/terrestrial lineages [55], [84]. All land crabs in Oceania have marine larvae. Such life history limits dispersion inland, and likely makes crabs more vulnerable to terrestrial enemies because of the predictable, mass migrations they undertake to the coast for larval release. However, marine larvae also allow colonization of islands, where absence of many predators allows greater levels of ecological experimentation, and thus may be conducive to the evolution of increased terrestriality.

Life histories independent of the sea had evolved on fewer occasions than terrestriality in crabs. Aside from the lineage(s) of ancient, diverse “freshwater crabs” (i.e. Potamoidea, Pseudothelphosoidea, and Trichodactyloidea; [85]), non-marine, abbreviated life histories are known in the freshwater, west Pacific Trogloplacinae, freshwater-terrestrial, Indo-Malayan *Geosesarma*, several species of cave-dwelling *Sesarmoides* in Indonesia, and a remarkable radiation of sesarmid crabs in Jamaica [26] [55], [75]. A noteworthy aspect of the Jamaican land crab radiation is that terrestriality evolved *in situ*, and only there within the Caribbean. Unlike other Greater Antilles, Jamaica is thought to have undergone partial or complete submergence, cleansing the island of most if not all continental taxa [86]. This example demonstrates importance of islands in the origin of terrestriality in crabs. Presumably because of the imperfection of their macroevolutionary transition onto land, land crabs are ecologically important mostly on, and many species are limited to, oceanic islands, where continental predators and competitors (e.g. mammals, large reptiles) are absent. However a consequence of the evolution of fully terrestrial/freshwater life histories, as in this Jamaican sesarmid radiation, is that it may restrict crabs to the island they evolved on, rendering them evolutionary dead ends from a global perspective.

Adaptations for semi-terrestrial existence may also make it difficult for crabs to return to subtidal habitats; alternatively, intertidal habitats may be a safe place for lineages that have not faired well in more diverse, and biologically intense, subtidal settings. We are struck by the lack of free-living, subtidal representatives in the major intertidal-terrestrial brachyuran clades. The closely related (and potentially not reciprocally-monophyletic) Grapsoidea and Ocypodoidea include most intertidal and land crabs outside the “freshwater crabs”. While these crabs are diverse, abundant, and dominate in intertidal and coastal habitats, they are scarce in subtidal habitats with a few exceptions: the deep water *Euchirograpsus*, hot-spring associated *Xenograpsus*, a few Varunidae, and some species of *Percnon* and *Macrophthalmus* (e.g., [29], [87], [88]). Furthermore, these superfamilies are united with pea crabs (Pinnotheroidea) and coral gall crabs (Cryptochiroidea), both symbiotic crab families that live within their host, into the Thoracotremata [55]. If the Thoracotremata is indeed monophyletic, it is remarkable that this diverse group has virtually no free-living shallow marine representatives. Intertidal, non-marine, deep water, and endo-symbiotic habitats are all “safe places” in the struggle for life, and may imply that survival (and diversification) of thoracotrematan crabs has been possible by their remarkable adaptability toward novel environment, rather than to an ability to compete in the face of biological pressures associated with the highly escalated shallow subtidal environment. Similar evolutionary lability, yet exclusion from “normal” shallow marine habitats are demonstrated by a few other taxa, for example the Neritomorpha among gastropods [89].

### Ecological roles of land crabs

The importance of land crabs in the terrestrial ecology of tropical islands, particularly atolls has long been recognized. As noted by Hedley (in [32]: 127–128, quoted in [63]): “The dominant note in the life of a coral atoll … struck me as the abundance and ubiquity of Crustacea. The Avifauna were but sea fowl, the indigenous Mammalia but rats, the Reptilia only a stray skink and gecko, while insects and land Mollusca … were barely represented. Into the vacant places swarmed Crustacea. Not an inch of the atoll world is secure from them.” The feeding habits of land crabs break down largely along taxonomic lines. Gecarcinids and many sesarmids are largely herbivorous/detritivorous (although with some tendency to omnivory, T. Naruse, pers. comm.), eating living and dead plant parts. Coenobitids (including *Birgus*) are omnivorous scavengers, feeding on vegetation, carrion, insects, etc. *Geograpsus* and the largely beach-restricted *Ocypode* are voracious carnivores/omnivores [52], [53], [76], [77].

Gecarcinids hold the record in terms of crab biomass per area, as they are large and often very abundant. On tiny Clipperton Island *Gecarcinus planatus* has reached population densities of 6 m^−2^ at times, literally reddening the island [90]. Although this represents the largest population density recorded for land crabs [91], it may not be uncommon in undisturbed settings. Clipperton is the only island we are aware that lacked all terrestrial mammals at the time land crab censuses were taken. Thus these numbers are indicative of the potential abundance of land crabs on islands prior to human influence. Nevertheless the abundance of crabs has changed markedly over time even on this ∼1.6 km^2^ island as a result of depredations by introduced mammals. Pigs were introduced in 1897 and proliferated, feeding heavily on the crabs. As a result Clipperton changed from virtually/completely unvegetated to having substantial plant cover, because the impact of crabs on the vegetation was reduced by pigs preying on crabs [79]. Removal of pigs in 1958 led to major increase in crabs and loss of vegetation, only to be reversed again with the recent introduction of black rats, which again led to a rapid decline of gecarcinids and concomitant rebound of vegetation [36].

Lower, but still large numbers of crabs are known from other islands that harbor some introduced mammals. On Christmas Island (Indian Ocean), the large (500 grams), endemic *Gecarcoidea natalis* occurs at densities of >1/m^2^, for an estimated population size of 100 million crabs [91]. They consume most accessible plant matter, including seeds, seedlings, and leaf litter, keeping the forest floor unusually clean and clear [91], [92], [93], [94], [95]. The loss of this crab from areas as a result of crazy ant invasion has lead to a doubling of litter cover, 30-fold increase in density and 3.5-fold increase in diversity of seedlings [35]. Gecarcinids are also important seed dispersers [96].

Several species of land crabs have been implicated as predators on bird chicks and eggs, and Atkinson [33] argues that land crabs may have made birds on tropical islands behaviorally more resilient to rats, than birds were on temperate islands, where land crabs are absent. Although a predominantly herbivorous omnivore [51], *Birgus* is known to prey on vertebrates, including tortoise and turtle hatchlings [53], [97] and even rats [98]. *Coenobita* have a broad diet, feeding on plants, carrion, insects and just about any available organic matter [53]. *Geograpsus* species are also important predators. They have the largest claws among the Grapsidae, well adapted to raptorial feeding. They prey on coenobitids, other land crabs (including congeners), insects and birds [52], [53], [54] (Fig. 12) (pers. obs.).

While sesarmids are relatively uncommon and/or small on Pacific islands, gecarcinids, *Birgus*, *Coenobita*, and *Geograpsus* are major ecological players in island ecosystems. However as noted above, the roles of these animals are quite different, ranging from largely herbivory with some omnivory (gecarcinids), through omnivory (coenobitids), to scavenging and carnivory (*Geograpsus*). While most islands in Oceania have all four taxa present, there is only reliable evidence for the prehistoric existence of *Geograpsus* in the Hawaiian Islands, and thus the role of crabs in Hawaii was likely limited accordingly. In this the Hawaiian Islands still differ from most other central Pacific islands, where gecarcinids and coenobitids are also abundant.

## Materials and Methods

**Figure 14:**
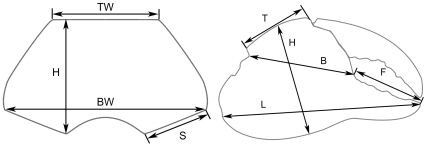
Measurements taken from chelae and sternites.

Fossil crab specimens were studied from collections assembled by Helen James & Storrs Olson (USNM), Mike Severns (Maui), David Burney (National Tropical Botanical Garden, Kaua'i), and their coworkers, and compared with the four known extant species of *Geograpsus* as represented in the collections of the American Museum of Natural History (AMNH), Bernice P. Bishop Museum, Honolulu (BPBM), Florida Museum of Natural History, University of Florida (UF), and US National Museum of Natural History (USNM). Chelae, carapace, and 4^th^ sternite (Fig. 14) were measured in selected specimens, to develop meristic criteria that discriminate species. Twenty discrete characters were also identified and coded in each *Geograpsus* species and in *Grapsus tenuicrustatus*, representing the putative sister genus [99]. Discrete character data were analyzed with PAUP v4.0, using maximum parsimony, with characters unweighted, character states unordered, with ACCTRAN character state optimization, treating multiple states as polymorphism, and running an exhaustive search. Bootstrap analysis with 10,000 replicates was used to evaluate clade robustness. Previous records of land crabs from the Hawaiian Islands were reviewed based on literature and selected specimen records.
